# A 38-year-old woman with necrotising cervical lymphadenitis due to *Histoplasma capsulatum*

**DOI:** 10.1007/s15010-017-1060-x

**Published:** 2017-08-18

**Authors:** Esther van de Vosse, Annelies van Wengen, Wendy F. van der Meide, Leo G. Visser, Jaap T. van Dissel

**Affiliations:** 1 Department of Infectious Diseases, Leiden University Medical Center, Room C5-42, Albinusdreef 2, 2333 ZA Leiden, The Netherlands; 2 U-CyTech Biosciences, Utrecht, The Netherlands

**Keywords:** IFN-γ, Autoantibodies, Histoplasmosis, HLA, Immunodeficiency

## Abstract

**Case presentation:**

We analysed a 38-year-old woman with disseminated histoplasmosis for primary immunodeficiency. Her blood showed no IFN-γ response while her peripheral blood mononuclear cells (PBMCs) did. We identified IFN-γ autoantibodies of the IgG class in her serum.

**Conclusion:**

IFN-γ autoantibodies leading to infections were so far mainly detected in people from Asian descent, where it was found to be associated with certain HLA types. This may be the first patient of African descent, and without the typical HLA types that predispose to this problem, that produces IFN-γ autoantibodies.

## Introduction

Autoantibodies against IFN-γ can lead to infections with opportunistic microbes, especially non-tuberculous mycobacteria. These autoantibodies directed against IFN-γ are thought to arise due to the 100% homology between peptides of the IFN-γ protein and the *Aspergillus* spp Noc2 proteins [1], which can be recognised by persons with certain haplotypes. A few HLA haplotypes have been found to be associated with the development of IFN-γ autoantibodies [2, 3], and these are mainly found in Asians, thus explaining why the IFN-γ autoantibodies are almost exclusively found in individuals from Asia or Asian descent [4–6]. We describe the first case of a patient from African descent that developed a severe opportunistic infection due to the presence of IFN-γ autoantibodies.

## Case presentation and discussion

**Fig. 1:**
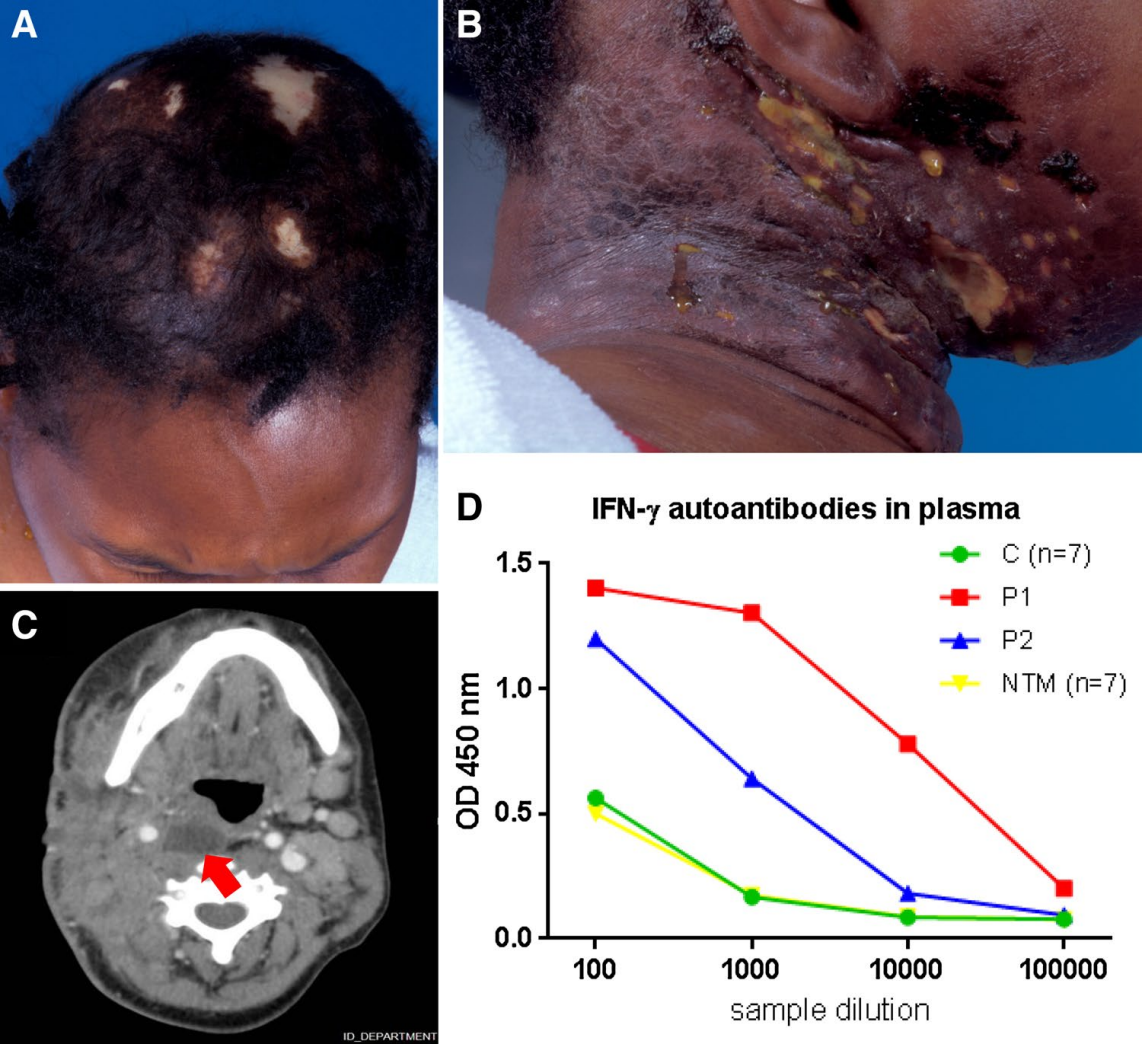
On clinical examination, alopecia (**a**) and multiple fistula of a necrotizing right-sided cervical mass (**b**) are noted. CT scanning of the neck (**c**) reveals an inflammatory mass from the mucosal space extending to the skin from the nasal pharynx all the way down to the upper thoracic aperture, with multiple abscesses including one located prevertebral (*arrow*). The right carotid artery and jugular vein are taken up in the mass, with the vein being compressed. *The left side* shows multiple enlarged lymph nodes. The graph (**d**) shows the presence of IFN-γ autoantibodies in plasma of seven healthy controls (C, mean), one patient with a high concentration of strongly inhibiting IFN-γ autoantibodies (P1), the current patient (P2), and seven patients with NTM infections (NTM, mean) as measured by ELISA. Plates were coated with IFN-γ and plasma was allowed to bind before detection of the anti-IFN-γ autoantibodies with HRP-labelled anti-IgG

We analysed a 38-year-old Surinamese woman with extensive cervical lymphadenitis and disseminated histoplasmosis for primary immunodeficiency. Her case report has been described before because of the remarkable presentation of the infection [7]. In short, she presented in early 2004 with a 4-month history of painful fistulating cervical mass, alopecia (Fig. 1), difficulty swallowing, fever, and weight loss. Her medical history included beta-thalassemia minor and blindness due to refractory glaucoma after panuveitis of both eyes. She is Surinamese from African descent. She immigrated to the Netherlands 10 years before first presentation and had last visited Suriname 3 years before presentation. On examination, an extensive cervical mass in the posterior triangle of her neck with firm induration and two draining fistulas, a shallow ulcer at the right side of the mouth, a pronounced swelling of the buccal mucosa (Fig. 1) and trismus was noted. CT scan of the neck showed extension of suppurative lymphadenitis into the retropharyngeal space (Fig. 1c). An X-ray of the thorax was normal; ultrasound of the abdomen revealed moderate hepatosplenomegaly. Laboratory examination revealed an erythrocyte sedimentation rate > 140 mm, hemoglobulin 5.5 mmol/l, MCV 77 fl, and leucocytosis 25.3 × 10^9^/l. She was HIV negative, had no MBL mutations and had normal CD4 + (920/mm^3^) and CD8 + (1028/mm^3^) lymphocyte counts. Normal numbers and percentages of B cells were present in blood and bone marrow. Leukocytes were increased initially and after that were stable at 4200/mm^3^. A lymph node biopsy revealed a necrotizing lymphadenitis with structures suggestive of yeasts. No granuloma was seen. Cultures of blood, abscess fluid and cervical lymph node biopsy grew *Histoplasma capsulatum*. The diagnosis of cervical lymphadenitis and chronic disseminated histoplasmosis was made. She received itraconazole 200 mg twice daily and reached adequate serum titres. After 2 weeks the fever subsided and she made a slow and steady recovery in the course of a year. Itraconazole was continued for another year and in a follow-up of 6 years there were no signs of recurrence.

There are several primary immunodeficiencies that predispose to invasive or chronic fungal diseases, such as defects in the fungal recognition pathway (DECTIN1, CARD9) and IL-17 signalling (a.o. in IL-17RA, IL-17F, STAT1) for *Candida* infections, and chronic granulomatous disease (CGD) and STAT3 defects predisposing to *Aspergillus* infections [8, 9]. Histoplasmosis on the other hand is found in patients with defects in the type 1 cytokine pathway—that are mainly known for conferring susceptibility to infections with mycobacteria and salmonellae [10–14]—as well as in defects resulting in combined immunodeficiencies that affect the presence of multiple cell types [8]. Apparently these different fungal pathogens are controlled or killed by different pathways.

Our working diagnosis was, therefore, a partial primary immunodeficiency affecting the type 1 cytokine pathway, such as a dominant-negative IFN-γR1 mutation, which would explain her infection as well as its late onset [13, 15]. To examine this we stimulated whole blood with various concentrations of lipopolysaccharide (LPS) and interferon (IFN)-γ to determine whether innate immune responses were affected. The patient’s blood cells showed repeatedly (2005, 2006, 2009) a good response to LPS, but little or no response to IFN-γ as determined by measuring IL-12p40, TNF, and IL-10 in the supernatants. When isolating peripheral blood mononuclear cells (PBMCs) from her blood we found normal IFN-γR1 expression on monocytes and that IFN-γ could induce STAT1 phosphorylation. Phosphorylated STAT1 was found to translocate to the nucleus upon stimulation with IFN-γ. Her *STAT1* gene was sequenced to rule out a mutation that would allow STAT1 phosphorylation, dimerization and subsequent translocation to the nucleus, but that would preclude binding of STAT1 to DNA; however, no mutation was found. On monocytes we also observed that IFN-γ could induce normal CD64 upregulation. This was an indication that a factor in the plasma was inhibiting the IFN-γ response. Using exchange experiments in which plasma of the patient was added to cells from controls and vice versa we were able to prove this was the case. Whole exome sequencing was performed to identify potential mutations but none were found.

At that time the first reports were published [16, 17] on an adult-onset immunodeficiency in which autoantibodies against IFN-γ were leading to infections with non-tuberculous mycobacteria (NTM) and other opportunistic bacteria and fungi [5], nearly completely restricted to individuals from Southeast Asia [6]. Although our patient was not of Southeast Asian descent her plasma was tested for the presence of antibodies against IFN-γ, these initial tests (on fresh samples in 2009) did not show it to contain more antibodies against IFN-γ than those of controls. Protein sequencing of the IFN-γ-binding fraction isolated from plasma did reveal antibodies. At a later time point (in 2014) inhibiting IFN-γ autoantibodies were detectable (U-CyTech bioassay), but at a concentration 200 lower, and a factor 16 less effective, than those in an Asian patient known to produce strongly inhibiting IFN-γ autoantibodies. Currently, IFN-γ autoantibodies in her plasma are readily detectable by ELISA (Fig. 1d), and these neutralizing IFN-γ autoantibodies were shown to be of the IgG class. The IFN-γ autoantibodies were shown in a bio-assay to block IFN-γ signalling (data not shown).

In a large study in patients with adult-onset immunodeficiency due to IFN-γ autoantibodies from Taiwan, limited HLA allele polymorphism was observed and HLA types A*11:01, B*40:01, DRB1*16:02, and DQB1*05:02 were found to be overrepresented [3]. In a follow-up study also including patients from Thailand the association with DRB1*16:02 and DQB1*05:02 was confirmed while also DRB1*15:02 was found to be associated [2]. In our patient none of her alleles were present homozygously. She had the HLA types: A*02/A*31, B*53:01:01/B*57, C*04:01:01/C*18:01, DQA1*01:02:01/DQA1*03:03:01, DQB1*02:02/DQB1*06, and DRB1*09/DRB1*13:02:01, so no overlap was seen with the HLA types in the previous studies [2, 3].

Patients with adult-onset immunodeficiency due to IFN-γ autoantibodies have been reported with disseminated fungal infections before. Disseminated histoplasmosis was found in seven of 97 patients from Thailand and Taiwan, while also other disseminated fungal infections, with *Cryptococcus neoformans* (in ten patients) and *Penicillium marneffei* (in seven patients), were found [5]. In 20 patients from Northern Thailand *Penicillium marneffei* was the most abundant disseminated fungal pathogen, while *Cryptococcus* and *Histoplasma* were also found [18]. No *Aspergillus* or *Candida* infections were reported in those two studies, confirming the observation that was made in patients with genetic defects that the control of various fungal pathogens requires different pathways. For the control of *Histoplasma* T cells, monocytes, and macrophages are the critical elements [13], while for the control of *Aspergillus* superoxide formation is essential and for the control of Candida the DECTIN1 and IL-17 pathways are essential. That not a larger proportion of patients with genetic defects in the type 1 cytokine pathway or with IFN-γ autoantibodies develop *Histoplasma* infections may have to do with the geographical distribution of *Histoplasma*. While NTM are ubiquitously present, *Histoplasma* has a geographically limited distribution as it is found mainly in soil that contains large amounts of bird or bat excreta [19].

One patient from South Africa, living in the UK, was reported with IFN-γ autoantibodies. Whether this patient was of African or Asian (or other) descent was not specified [20]. Our patient is, therefore, the first patient from definite African descent who was found to produce IFN-γ autoantibodies. Including our patient, in total five patients have now been reported that developed IFN-γ autoantibodies that were not of Asian descent [4, 20]. It is important to remember that although a patient does not have an Asian background, IFN-γ autoantibodies that cause opportunistic bacterial or fungal infections may nevertheless be present. The autoantibodies in this patient were effectively inhibiting IFN-γ signalling but due to their low concentration at onset these were initially missed. One should keep in mind that patients that develop adult-onset immunodeficiency due to autoantibodies against IFN-γ may have a period in which the autoantibodies are not present at a high concentration yet but are nevertheless clinically relevant.
